# Knowledge, Attitudes, and Perceived Barriers to Genetics and Precision Medicine Among Rural Participants in a Large Academic Biobank: A Mixed-Methods Study

**DOI:** 10.3390/jpm16090446

**Published:** 2026-08-26

**Authors:** Meghan MacNeal, Nathan A. Bihlmeyer, Susanne B. Haga

**Affiliations:** 1 Center for Precision Health, Duke University School of Medicine, Durham, NC 27710, USA; meghan.macneal@duke.edu; 2 Clinical Research Unit, Department of Medicine, Duke University School of Medicine, Durham, NC 27710, USA; nathan.bihlmeyer@duke.edu; 3 Division of General Internal Medicine, Department of Medicine, Duke University School of Medicine, Durham, NC 27710, USA

**Keywords:** rural, attitudes, precision medicine, genetics knowledge

## Abstract

**Background/Objectives:** Rural populations remain underrepresented in genomic research and may face unique barriers to accessing genetic and precision medicine services. This study evaluated knowledge, attitudes, beliefs, and perceived barriers related to genetics and precision medicine among rural participants enrolled in OneDukeGen (ODG), a large academic biobank and precision medicine research initiative. **Methods:** This mixed-methods study included rural ODG participants identified using U.S. Census Bureau rural classification code. Participants completed an online survey assessing genetics knowledge, attitudes toward genetic testing and precision medicine, healthcare utilization, and internet use. Semi-structured interviews were conducted with a subset of survey participants to further explore perceptions of genetics research, barriers to care, privacy concerns, and educational needs. **Results:** Among 10,305 ODG participants, 3268 (31.7%) were classified as rural, representing 97 of North Carolina’s 100 counties. A total of 111 rural participants completed surveys and 14 participated in qualitative interviews. Participants generally demonstrated favorable attitudes toward genetics and precision medicine despite moderate genetics knowledge scores. Most participants believed genetic testing could improve disease prevention and treatment selection, particularly for cancer care and pharmacogenomics applications. Qualitative interviews identified three major thematic domains: (1) perceived value and promise of genetics, (2) concerns regarding privacy, trust, and misuse of genetic information, and (3) barriers and facilitators to accessing genetic services. Participants generally viewed genetics research positively but expressed concerns regarding cost, insurance coverage, privacy, and genetic discrimination. **Conclusions:** Rural participants generally expressed positive attitudes toward genetics and precision medicine; however, substantial barriers related to cost, trust, privacy, awareness, and digital access remain. Community-engaged approaches emphasizing accessibility, provider education, and culturally appropriate communication may be critical for equitable implementation of precision medicine in rural communities.

## 1. Introduction

Rural populations are underrepresented in biomedical research [1,2,3,4] and face barriers to accessing specialty healthcare services [5], including clinical genetics and precision medicine services [6]. Although definitions of rurality vary, rural areas are generally characterized by lower population density and greater geographic dispersion. Rural communities may experience healthcare access barriers related to geographic distance from academic medical centers, limited availability of genetic counseling services, and disparities in digital connectivity and healthcare infrastructure. Understanding rural populations’ knowledge, attitudes, and perceptions regarding genetics and precision medicine is important for the equitable implementation of genomic healthcare and the development of community-engaged research initiatives.

Health disparities in rural populations are well-documented and include higher rates of chronic disease, poorer health outcomes, and reduced access to healthcare services compared with urban populations [7,8,9]. According to the latest statistics from the US Census Bureau, about 20% of the US population resided in a rural area in 2020 [10]. North Carolina has a substantially larger rural population, with approximately 33% of residents living in rural areas [11]. In North Carolina alone, 3,474,661 people lived in rural areas as of the 2020 Census, representing the second-largest rural population in the U.S., behind only Texas [10,11]. These communities may experience barriers related to transportation, healthcare workforce shortages, insurance access, and broadband availability, all of which may influence access to genetic testing and precision medicine applications.

OneDukeGen (ODG) [12] is a large academic biobank and precision medicine research initiative designed to study the role of genetics in health and disease and return selected medically actionable genetic results to participants. Approximately one-third of ODG participants reside in rural areas, providing an opportunity to better understand perceptions of genetics and precision medicine among rural research participants across North Carolina.

Prior studies have suggested that rural populations may demonstrate generally favorable attitudes toward genomic medicine despite variable levels of genetics knowledge [13,14,15,16,17,18]. However, concerns related to privacy, cost, trust, and healthcare access may influence willingness to participate in genomic research or pursue clinical genetic services. This mixed-methods study evaluated rural participants’ knowledge and attitudes toward genetics and precision medicine, identified perceived barriers to accessing genetic services, and explored educational and clinical support needs. These findings may help inform the development of more accessible and community-engaged approaches for implementing precision medicine in rural populations.

## 2. Materials and Methods

We conducted a mixed-methods study to explore rural residents’ perspectives on (1) their knowledge and attitudes regarding genetics, genomics, and precision medicine, and (2) the clinical tools and resources they believe could enhance access to precision health services. The study consisted of two phases: (1) an online survey and (2) a semi-structured phone interview. The study was approved by the Duke University Health System Institutional Review Board.

**Figure 1:**
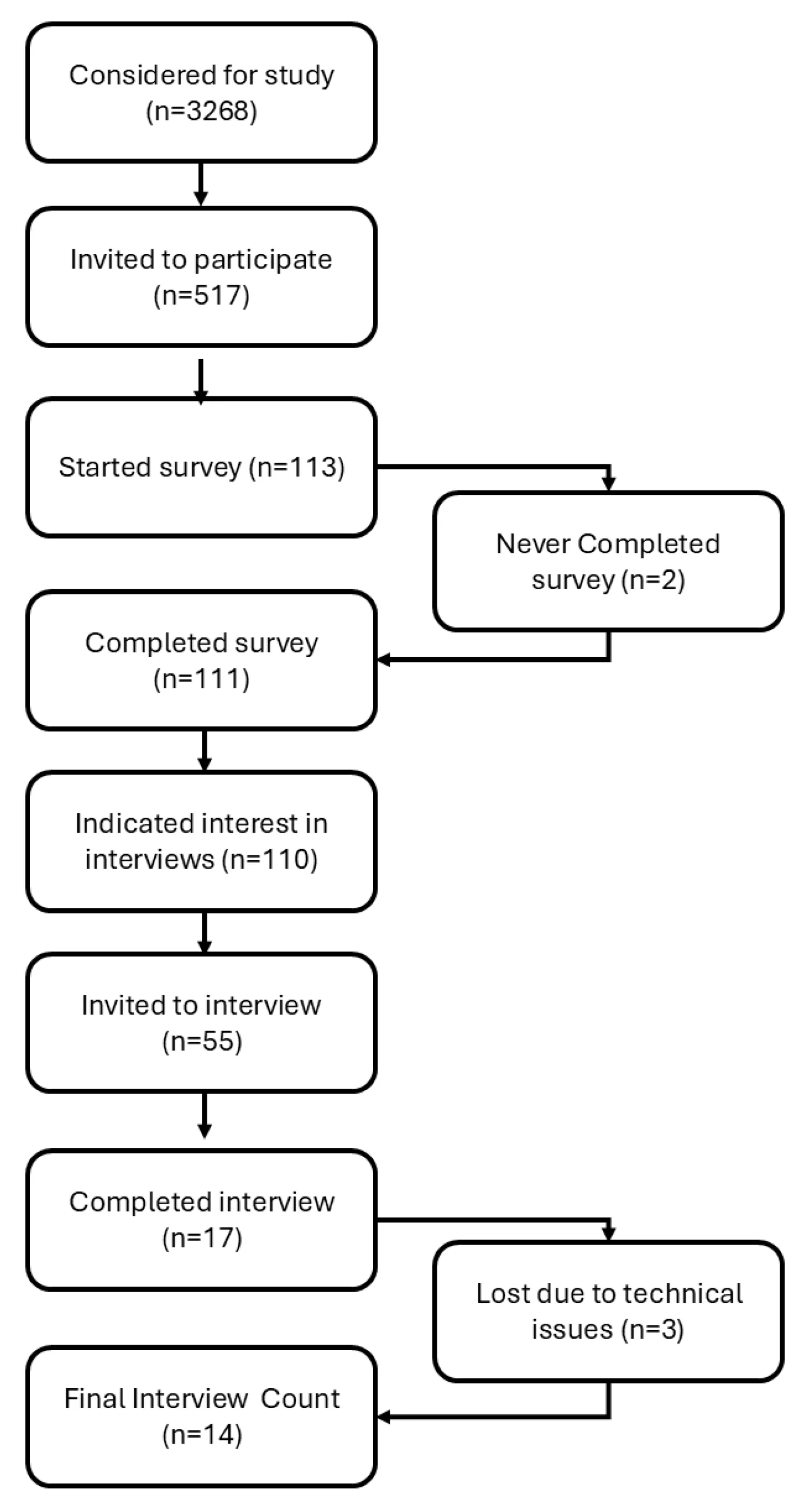
Recruitment diagram for survey and interviews.

Target Population. A total of 3268 rural participants were identified in the ODG study population on 16 May 2025. ODG is a research study that aims to discover how genetics contributes to health and disease, improve prevention and treatment, and return certain medically actionable results to participants. Participants provide a saliva or blood sample for genetic sequencing, complete online surveys about their health and family history, and allow access to their medical records. ODG participants are recruited from various clinics at Duke University Health System and can enroll in-person or remotely. Rural participants were identified using the 2020 Primary Rural-Urban Commuting Area (RUCA) codes from the U.S. Census Bureau [19]. Participants with a missing or incomplete email address or who have passed away since enrollment were removed from the list. A random group of 517 participants was invited to participate in the survey study (Figure 1).

Survey. An online survey was created with a total of 75 questions. Questions were adapted from published surveys on genetics knowledge, healthcare utilization and barriers to access. Questions regarding knowledge of genetics and genomics were adapted from the PUGGS questionnaire [20]. Questions regarding healthcare access and utilization were adapted from the All of Us Healthcare Access and Utilization survey [21]. Questions regarding internet connectivity and devices were adapted from the Pew Internet/Broadband survey [22]. Demographic data were obtained from the enrollment survey in OneDukeGen. The survey was developed and administered through RedCap. A study invitation and one follow-up reminder were emailed to participants.

Interviews. At the conclusion of the survey, participants could indicate their interest in participating in the interviews. An interview guide of seven main questions was developed to gather more information regarding participants’ views on awareness and attitudes of genetics research and clinical genetic applications and clinical support needs. A total of 110 survey respondents indicated interest in participating in the semi-structured interviews. We invited a random sample of 55 participants to schedule an interview. A total of 17 participants completed the interview; recordings from three interviews were lost due to technical issues and were therefore unavailable for analysis, yielding a total of 14 interviews. The interview session lasted approximately 30 min. All interviews were conducted by phone, audio-recorded with participant consent and transcribed.

Data Analysis. For the survey data, we utilized both descriptive and inferential statistical techniques to determine patterns and relationships within the collected responses. Demographic characteristics of participants and their responses to survey items were first summarized using descriptive statistics, including frequencies, means, and standard deviations. To identify potential associations between variables, cross-tabulation and chi-square tests of independence were performed. For Likert scale data, Fisher’s Exact Test was implemented to assess associations where expected cell counts were low. To analyze attrition at each study stage, chi-square or *t*-tests compared participants who progressed to the next step against those who dropped out (e.g., comparing survey completers vs. non-completers among those invited to participate, and interviewees vs. non-interviewees among survey completers). To test whether beliefs regarding genetic determinism differ across educational levels, we used linear mixed-effects models (LMM). The dependent variable was the participant response across the 16 genetic traits. To account for within-participant correlation caused by these repeated measures, we included a random intercept for each participant. We evaluated the fixed effect of education level by performing a likelihood ratio test (via ANOVA) comparing a baseline model (containing only the question type and the random intercept) to an alternative model that added education level as a fixed factor. These analyses enabled us to draw conclusions about the broader population and support the development of targeted interventions based on the identified needs and trends. All statistical analyses were conducted using R version 4.6.1. [23].

For the semi-structured interviews, transcripts were analyzed independently by both authors using an inductive thematic analysis approach [24]. Initially, each author reviewed the transcripts multiple times to achieve familiarity with the data and identify preliminary codes, recurring concepts, and emerging themes. Codes and themes were generated inductively, allowing patterns to emerge directly from the data rather than being constrained by predefined categories or hypotheses. The authors subsequently compared coding interpretations and collaboratively refined thematic categories through iterative discussion to achieve consensus regarding the major themes and subthemes identified across interviews. Discrepancies in coding or interpretation were resolved through discussion and re-review of the original transcripts to ensure that findings remained grounded in participants’ responses. A second round of transcript analysis was conducted using the finalized coding framework, and representative quotations were selected to illustrate the major themes and subthemes. During analysis, the later interviews yielded no substantially new themes, suggesting that thematic saturation had been achieved for the principal domains explored.

## 3. Results

ODG Participants. As of 16 May 2025, a total of 10,305 participants were enrolled in the OneDukeGen (ODG) study and 31.7% resided in a rural area. Overall, the ODG population was predominantly White (85.8%), female (51.2%), and older, with the largest age group between 60–74 years (45.8%) (Table 1). Compared with North Carolina census data for rural residents, the demographic distribution of the ODG cohort was generally similar with respect to sex, but differed significantly by race and ethnicity, with lower enrollment of African American and Hispanic participants.

Statistical tests were also performed to explore demographic characteristics of ODG study participants compared to participants in the All of Us Study cohort [25] and Duke University study cohorts (all protocols listed in the Duke Clinical Research Management System) [26] (Table 1). A total of 3268 ODG participants (31.7%) were identified as residing in rural areas based on U.S. Census Bureau rural classification, the majority residing in North Carolina (n = 2715; 83.1%), representing 97 of the 100 counties in North Carolina. The proportion of rural participants in ODG exceeded the proportion of rural participants reported in the NIH All of Us Research Program, where only 3.1% of participants were classified as rural using a healthcare access-based proxy measure. In contrast, the rural representation observed in ODG was substantially higher than the approximately 19% rural participation reported in Duke CTSI clinical research populations using RUCA-based classifications.

Among rural participants, ODG demonstrated significantly lower representation of African American participants and females and higher representation of White participants, as well as significantly fewer Hispanic participants compared to the other two cohorts.

Survey and Interview Study Populations. A total of 517 rural ODG participants were invited to participate in the study, and 111 completed the online survey (response rate: 21.5%) (Figure 1). Of the survey respondents, 58.6% were female, the median age was 63 years, and the majority identified as White (87.4%) (Table 2). Most respondents had completed at least some college education (85.6%), and 43.2% were retired. Medicare represented the most common form of insurance coverage (57.7%), followed by private insurance (37.8%). Most respondents (82.0%) had enrolled in ODG remotely using a mailed home collection kit. More than half of survey respondents (55.9%) reported previous personal experience with genetic testing.

Among survey respondents, 110 individuals expressed interest in participating in interviews. A random subset was invited, and 17 interviews were completed. Due to technical recording issues, three interviews were unavailable for analysis, resulting in a final qualitative sample of 14 interviews. Interview participants were evenly divided by sex, predominantly White (85.7%), and had a median age of 68 years.

**Figure 2:**
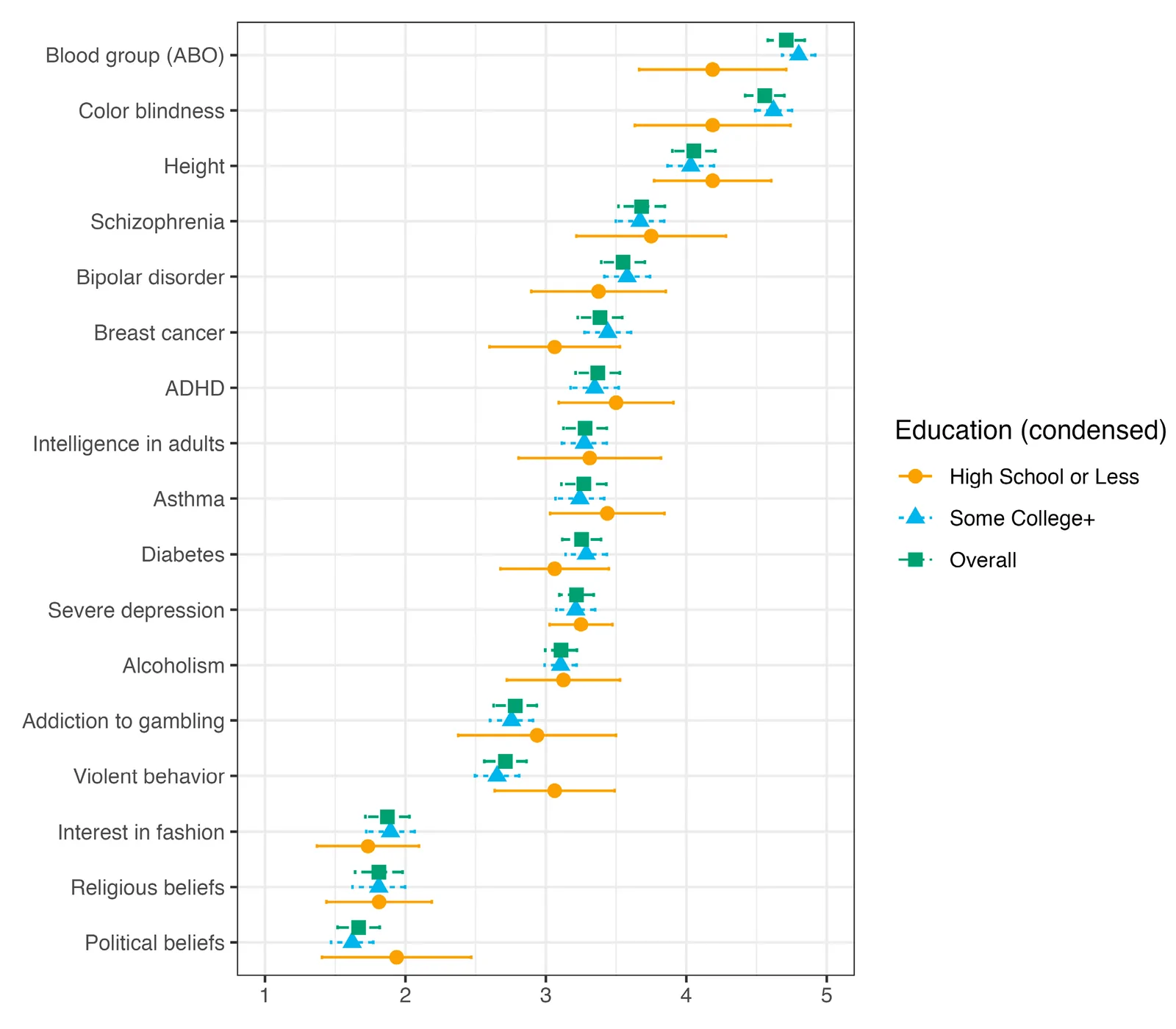
Results of survey responses to questions regarding genetic vs. environmental contributions to various traits and conditions.

Beliefs in Genetic Determinism. Participants were asked to rate the extent to which 16 traits or conditions were influenced by genetics versus environmental factors using a 5-point scale (1 = only environmental; 5 = only genetic). Overall ratings ranged from 1.7 to 4.7, with a mean rating of 3.2 across all traits, suggesting that respondents generally viewed most health conditions and traits as influenced by both genetics and environmental factors (Figure 2).

Traits most strongly perceived as genetically determined included blood type (4.7), color blindness (4.6), and height (4.0). In contrast, social and behavioral characteristics such as political beliefs (1.7), religious beliefs (1.8), and interest in fashion (1.9) were viewed as predominantly environmentally influenced. Ratings for most medical and psychiatric conditions, including diabetes, asthma, breast cancer, schizophrenia, and severe depression, fell within the intermediate range (3.1–3.7), reflecting beliefs that both genetic and environmental factors contribute to disease risk. No significant differences in overall beliefs regarding genetic determinism were observed by sex (*p* = 0.71) or education level (*p* = 0.78).

**Figure 3:**
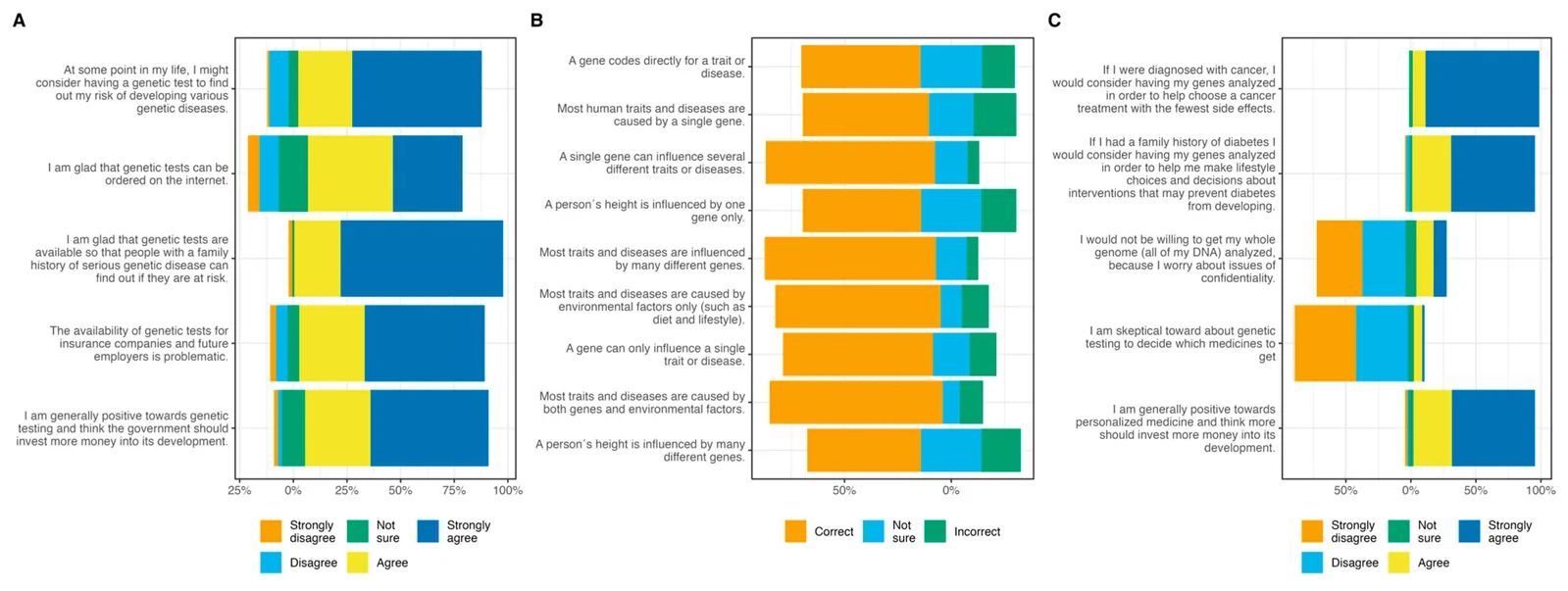
Results of survey responses to questions on attitudes about genetic testing. (**A**) respondents’ level of agreement with statements related to attitudes toward genetic testing; (**B**) % correct on knowledge survey regarding genetics and genomics; (**C**) respondents’ level of agreement with statements related to precision medicine and test utilization.

Attitudes toward Genetic Testing. Survey respondents generally expressed favorable attitudes toward genetic testing. The majority indicated that they would consider genetic testing in the future to assess disease risk, with 60.3% strongly agreeing and 25.2% agreeing with this statement (Figure 3A). Similarly, 75.7% strongly agreed that genetic tests are beneficial for individuals with a family history of serious genetic disease.

Participants also demonstrated strong support for continued investment in genetics research and testing, with 55.0% strongly agreeing that the government should invest more money into the development of genetic testing technologies. However, attitudes toward direct-to-consumer testing were somewhat more cautious. Although most participants remained positive regarding internet-based access to testing, 13.5% reported uncertainty regarding online genetic testing availability. Concerns regarding privacy and misuse of genetic information were also evident, as 55.9% strongly agreed that access to genetic testing results by insurance companies or employers could be problematic.

Participants with prior genetic testing experience were significantly more likely to report willingness to undergo future testing for disease risk assessment (*p* = 0.02) and stronger agreement that genetic testing benefits individuals with a family history of serious disease (*p* = 0.04).

Genetic Knowledge. Overall knowledge regarding genetics and genomics was moderate. Across nine true/false knowledge questions assessing understanding of genes and environmental influences on disease, respondents achieved a mean score of 6.10 out of 9 (67.8%) (Figure 3B). The statement “Most traits and diseases are caused by both genes and environmental factors” had the highest percentage of correct responses (81.1%), indicating broad recognition of multifactorial inheritance. Respondents also demonstrated relatively strong understanding that many diseases are influenced by multiple genes (80.2%) and that a single gene may influence multiple traits or diseases (79.3%). However, misconceptions remained regarding polygenic inheritance and complex traits; only 53.2% correctly identified that height is influenced by many different genes. Knowledge scores did not differ significantly by sex (*p* = 0.23), educational level (*p* = 0.17), or prior experience with genetic testing (*p* = 0.42).

Attitudes Toward Precision Medicine and Test Utilization. Participants generally expressed positive attitudes toward precision medicine applications. The highest level of agreement was observed for the use of genetic testing to guide cancer treatment selection and reduce treatment side effects, with 86.9% strongly agreeing with this application (Figure 3C). Participants also expressed support for the use of genetic testing to guide prevention and management strategies for chronic disease, including diabetes. Although respondents generally supported personalized medicine and pharmacogenomics, some participants also expressed concerns regarding privacy and the possibility of obtaining unrelated genetic findings through genome sequencing. Participants with previous genetic testing experience were significantly more likely to strongly support the use of genetic testing to guide cancer treatment (*p* = 0.003) and diabetes management decisions (*p* < 0.001).

Health Status and Utilization. Overall, 42.3% of respondents rated their health as “good,” while 18.9% reported that their health had worsened during the previous year. Respondents reported relatively frequent healthcare utilization, with 21.6% indicating 16 or more healthcare visits in the previous 12 months. Regarding annual healthcare visits, significant differences were observed between sexes (*p* = 0.02). Females exhibited a bimodal distribution, with peaks at the lowest (≤5 visits; 33.8%) and highest (≥16 visits; 27.7%) ends of the spectrum. In contrast, male utilization peaked in the 6–9 visit range (50%). The disparity was most pronounced at the upper extreme, where females were more than twice as likely as males to report 16 or more visits (27.7% vs. 13.0%).

Barriers to care. A substantial proportion of respondents (42.3%) reported delaying healthcare within the previous year. The most common reasons included out-of-pocket healthcare costs (15.3%), nervousness about seeing a healthcare provider (11.7%), and travel distance to healthcare providers (11.7%). In addition, 21.6% reported declining prescribed medications because of cost (Table 3).

Information Seeking/Social Media. Healthcare providers represented the primary source of health information for most respondents (86.5%). Internet access and technology use were common within the study population, with all respondents reporting ownership of a cell phone and most reporting access to either a home computer (81.1%) or tablet device (64.9%). Most respondents used the internet several times daily (59.5%), while an additional 34.2% reported using the internet almost constantly.

**Figure 4:**
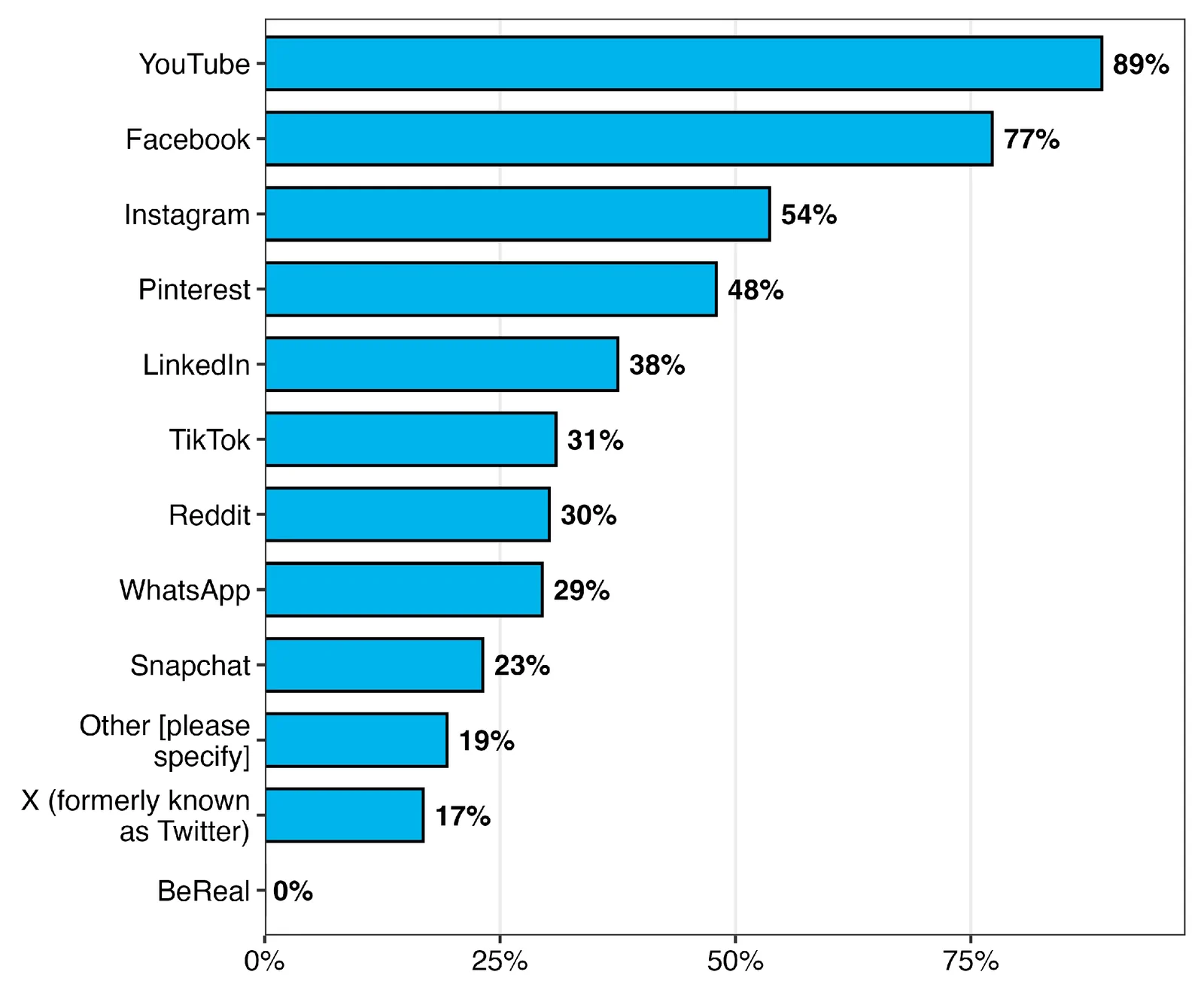
Survey respondents’ social media usage.

The majority of respondents (86.5%) also reported searching online for health information. Government or hospital websites were the most frequently utilized online sources (63.6%), followed by other general websites (59.8%). Social media platforms were less commonly used for health-information seeking (8.4%); Facebook and YouTube were the most frequently identified social media platforms when used (Figure 4).

Interviews. Thematic analysis of the semi-structured interviews identified several themes related to rural residents’ perceptions of genetics research, clinical genetic applications, and barriers to accessing genetic services (Table 4). Themes were organized into four overarching domains: (1) perceived value and promise of genetics; (2) concerns regarding trust, privacy, and misuse; (3) education and outreach; and (4) barriers and facilitators to accessing genetic services.

### 3.1. Perceived Value and Promise of Genetics

Participants broadly viewed genetics research and clinical genetic testing as promising and potentially transformative for healthcare. Many associated genetics with scientific progress, improved disease prevention, personalized medicine, and more effective treatments. Participants frequently described genetics research as “fascinating,” “helpful,” and “important for the future.”

Several participants emphasized the potential for genetics to improve medication selection and reduce ineffective “trial-and-error” prescribing approaches. One participant explained: “If my genetic makeup suggests that one kind of medicine would be really effective for me and another kind wouldn’t, then that has great potential” [73-year-old White male]. Others described genetics as valuable for identifying inherited disease risks, such as cancer, and enabling earlier interventions. Participants also expressed interest in genetics as a tool for understanding family history and inherited disease susceptibility. Some participants connected genetics to broader concepts of preventive medicine and individualized healthcare. One participant highlighted the importance of genetics in mainstream healthcare: “I think it’s something that should be part of like your regular healthcare” [51-year-old female, multiple races].

Relevance of Genetics to Rural Communities. Most participants believed genetics research and clinical applications were relevant to rural communities despite acknowledging that awareness and utilization may currently be limited. Participants often emphasized that the potential benefits of genetics were universal and not confined to urban populations, with one participant noting, “we have to get support in every community because…genetic disorders don’t discriminate” [38-year-old White female] and another suggested “Well, I mean, I think just that area of science can be helpful to anybody anywhere” [50-year-old White female]. However, some participants noted that rural residents may be less likely to pursue testing due to skepticism, limited exposure to genetics education, or concerns regarding privacy and outside institutions. One participant noted that some people in their community might not pursue genomics healthcare as “some people here are just not open to science” [59-year-old female, multiple races].

### 3.2. Privacy, Confidentiality, and Data Security Concerns

Although participants generally supported genetics research, concerns regarding privacy and misuse of genetic information were common. Participants frequently expressed uncertainty regarding how genetic information could be used in the future, particularly by governments, insurance companies, law enforcement, or commercial entities. Participants discussed concerns about government overreach, unauthorized data access, cyber threats, genetic surveillance, and misuse of DNA databases. Some mentioned concerns regarding future misuse by “the wrong hands.” While some participants acknowledged these concerns as hypothetical, they nevertheless viewed them as important concerns. For example, one participant acknowledged the conflict: “I have like two conflicting thoughts at the same time like on one hand I think it can open up so many doors to the future in medicine. As far as like new treatment and just learning more about isolating, different genes and stuff like that, but then on the other hand, I feel like it could also be scary like used in the wrong hands are in the wrong way” (50-year-old White female). Another participant commented it was inevitable “that all the government going to get his DNA and they gotta do this and they’re gonna do that with it” (50-year-old female, multiple races).

Concerns About Genetic Discrimination. Concerns regarding insurance discrimination and the potential use of genetic information against patients were also prominent. Some participants worried that genetic testing results could affect insurance eligibility or coverage decisions. One participant stated: “Insurance was like this is a pre-existing condition… that would be my only concern” [50-year-old White female]. Participants frequently compared genetic information to other highly sensitive medical information such as HIV status, mental health treatment, or substance use history.

Distrust of Commercial Genetic Testing Companies. Participants consistently differentiated between academic medical institutions and commercial genetic testing companies. Duke, UNC, NIH-affiliated research, and healthcare systems were generally viewed as trustworthy due to perceived ethical oversight, HIPAA protections, and institutional accountability. In contrast, participants expressed greater skepticism toward direct-to-consumer genetic testing companies such as 23andMe and Ancestry. One participant explained: “I haven’t done it because I’m afraid of what companies like that will do with my DNA” [50-year-old White female] and another noted that there is “no guarantee of privacy, even though that I read their privacy statements and stuff and we’re seeing some play out of that now the database is being sold [69-year-old White male].” Participants frequently framed trust as the determining factor influencing willingness to participate in genetic testing or research.

### 3.3. Education and Outreach

Limited Awareness and Knowledge About Genetic Services. Despite generally favorable attitudes toward genetics, many participants reported limited understanding of available genetic services or how to access them. Participants commonly stated they did not know where testing was available, what testing involved, how results would be interpreted, or whether testing would be clinically useful. Several participants noted that healthcare providers had never discussed genetic testing with them. One participant commented “No doctor has ever talked to me about genetic testing with all my conditions” [50-year-old White female]. Another participant noted “People rarely question medical testing per se, but part of me thinks that when you start talking about genetics, then that may change a little bit, I just don’t know” [69-year-old White male].

Awareness and Information Needs. Many participants reported limited awareness regarding how genetic services are accessed clinically, where testing is available, or how genetic results would be interpreted. Participants strongly endorsed increased education and community outreach related to genetics and precision medicine. Among the types of information desired by participants regarding genetic testing were what genetic testing entails, what conditions are evaluated, privacy protections, costs, and how results would affect care. Participants repeatedly highlighted the importance of trusted local institutions in facilitating engagement with genetics-related healthcare. Rural participants often described their communities as cautious, private, and skeptical of unfamiliar systems or outside authorities. One participant noted “People are much more guarded… there’s a cultural piece to that” [50-year-old White female].

Information Delivery. With respect to how information should be delivered, many participants emphasized the importance of utilizing multiple communication channels rather than relying on a single method. One participant stated that they would “…start with my doctor and also I will, you know, look online …you know, Google and search engine” [73-year-old White male]. Suggested outreach formats included printed materials (e.g., brochures, waiting-room materials), online videos (e.g., webinars), and in-person communications (e.g., health fairs, physician discussions, and community meetings). However, online materials may not be accessible to all. One participant noted “… there are a lot of people in this area who have zero Internet access. So that can be really hard so they’d have to get to like a library” [50-year-old White female].

In addition, some participants emphasized the importance of plain-language educational materials and practical explanations of the testing process. Trusted institutions included primary care providers, local clinics, churches, Veterans’ healthcare systems, schools, and community organizations. In particular, churches and church leadership were identified as trustworthy sources in some rural communities. Participants suggested that community-endorsed educational events could also improve acceptance and engagement, with one participant noting “I think you need a representative like to go out and talk” [54-year-old white female].

### 3.4. Barriers to Care

Participants identified several barriers to accessing clinical genetic services, including cost, insurance uncertainty, limited awareness of available services, and, for some participants, geographic and technological limitations associated with rural residence. Perspectives regarding travel and transportation barriers varied. Some participants reported relatively easy access to large academic medical centers such as Duke, UNC, or VA-associated facilities, while others described challenges related to rural infrastructure and the lack of nearby specialized services.

Participants also expressed mixed perspectives regarding telehealth and internet access. Perspectives regarding digital access varied substantially across participants and communities. Some described internet access as widely available and telehealth as convenient while others noted substantial broadband limitations and limited familiarity with digital technologies. One participant commented, “There are definitely a lot of areas we just don’t have any internet access” and “I think a lot of people, like they’re not as used to using electronic communication maybe than people in more urban areas” [50-year-old White female]. 

Another commonly identified barrier to accessing clinical genetic services was cost. Participants frequently expressed uncertainty regarding insurance coverage and concern that genetic testing would primarily benefit wealthier individuals. One participant stated:

“…things like gene editing for a particular medical treatment or things like that…. that a great potential would also have great expense and that regular people wouldn’t be able to afford them or their insurance wouldn’t cover them, so it’s likely that the primary or initial beneficiaries will be wealthy people.” [73-year-old White male].

Others described insurance coverage as the “main barrier” to access. Participants emphasized that even when interested in testing, uncertainty regarding affordability reduced willingness to pursue services. One participant mentioned “I didn’t think my insurance cover it so I didn’t think I could do it or afford it because it was said that it would have to be paid out-of-pocket or something” [59-year-old female, multiple races]. Additional barriers included lack of knowledge regarding where to obtain genetic services, uncertainty about the testing process, and concerns regarding privacy and potential insurance discrimination.

## 4. Discussion

The equitable implementation of genetics and precision medicine depends not only on advances in genomic technologies, but also on patients’ understanding, trust, and ability to access genomic services. Although rural populations have historically experienced disparities in healthcare access and remain under-represented in genomic research, relatively little is known about their perspectives on precision medicine. In this mixed-methods study of rural participants enrolled in OneDukeGen, respondents generally expressed favorable attitudes toward genetics and precision medicine despite only moderate genetics knowledge. At the same time, survey and interview findings consistently identified cost, insurance uncertainty, privacy concerns, limited awareness of available services, and healthcare access as important barriers. Together, these findings suggest that willingness to engage with precision medicine among rural populations may depend less on scientific understanding than on trust, accessibility, and confidence that genomic services are affordable, clinically useful, and responsibly managed.

An important strength of this study is the broad representation of rural participants within the OneDukeGen cohort. Nearly one-third of enrolled participants resided in rural areas, representing almost every county in North Carolina. Compared with both the NIH All of Us Research Program [25] and Duke CTSI clinical research cohorts [26], OneDukeGen enrolled a substantially higher proportion of rural participants. This difference likely reflects the study’s partly decentralized recruitment model, including remote enrollment, which may reduce logistical barriers that often limit research participation in rural communities. These findings demonstrate that thoughtfully designed recruitment strategies can improve engagement of geographically dispersed populations and suggest that under-representation of rural residents in genomic research is not inevitable. Because participants were already enrolled in a Duke-affiliated research initiative, trust in Duke Health and interest in biomedical research may also have contributed to participation.

One of the most notable findings was the disconnect between participants’ moderate genetics knowledge and their overwhelmingly positive attitudes toward genetic testing and precision medicine. Participants recognized the potential of genetics to improve disease prevention, treatment selection, and personalized care even though misconceptions regarding complex inheritance remained. Similar observations have been reported in other studies of genomic medicine [27,28,29,30], where favorable attitudes frequently coexist with limited genomic literacy. Anderson et al. [18] reported high expectations among patients undergoing genomic tumor testing despite limited understanding of genomic concepts, while Davies et al. [15] similarly found that participants viewed molecular profiling favorably despite low knowledge levels. While differences in genetics knowledge have been reported between rural versus urban patient populations [14,31], it is not always significant when adjusted for education and income level [14]. Other surveys of rural communities have reported awareness of genomic testing for cancer screening and genetic counseling, respectively, and very positive attitudes about recent genetic advances and counseling [32,33]. A narrative review of patient understanding of tumor multigene sequencing also concluded that positive attitudes toward genomic testing frequently coexist with substantial knowledge gaps [13]. Therefore, rather than suggesting that detailed genomic knowledge is a prerequisite for engagement knowledge [16], our findings indicate that individuals may evaluate precision medicine primarily through its perceived clinical relevance and potential health benefits. This distinction has important implications for implementation efforts because educational interventions should emphasize practical applications, realistic expectations, and informed decision-making rather than attempting to substantially increase genomic literacy alone.

The qualitative interviews further demonstrated that rural communities should not be characterized as resistant to genetics or precision medicine. Instead, participants generally viewed precision medicine as relevant and beneficial while acknowledging that many community members remain unfamiliar with available services or uncertain about how to access them. Participants frequently described their communities as cautious, private, and skeptical of unfamiliar institutions rather than opposed to science itself. This distinction suggests that barriers to implementation are largely structural and relational rather than ideological. Consequently, implementation strategies that rely on trusted local healthcare providers, community organizations, and other established institutions may be considerably more effective than approaches focused solely on increasing public awareness.

Trust emerged as a central theme across both the survey and interview data. Participants expressed concerns regarding privacy, cybersecurity, insurance discrimination, and potential future misuse of genetic information, yet these concerns rarely translated into outright rejection of genomic research or clinical applications. Although the federal Genetic Information Nondiscrimination Act (GINA) prohibits discrimination based on genetic information in health insurance and employment, these protections do not extend to life, disability, or long-term care insurance. The persistence of concerns regarding genetic discrimination among participants may reflect limited awareness of existing protections, concerns about gaps in those protections, or both. Respondents consistently distinguished between academic healthcare institutions and commercial direct-to-consumer genetic testing companies, expressing substantially greater confidence in university- and hospital-based research programs because of perceived oversight, transparency, and accountability. This finding reinforces previous research demonstrating that institutional trust is a critical determinant of participation in genomic research and suggests that communication efforts should emphasize privacy protections, governance, and responsible stewardship of genetic information in addition to the clinical value of testing.

Although participants were enthusiastic about precision medicine, they also identified practical barriers that may limit equitable implementation. Financial concerns emerged more consistently than geographic barriers, aligning with findings reported by other studies [34]. Survey respondents frequently delayed healthcare because of costs, while interview participants expressed uncertainty regarding insurance coverage and concerns that genetic testing would primarily benefit individuals with greater financial resources. Geographic distance and transportation challenges were acknowledged but appeared secondary to affordability and awareness. These findings suggest that expanding precision medicine in rural communities will require more than increasing the availability of genetic services. Policies that improve insurance coverage, reduce out-of-pocket costs, and clearly communicate financial expectations may be equally important for promoting equitable access. Other studies of rural communities about genomic screening for cancer reported that access and geographic proximity to testing [35] and no access to genetic counselors to review the results with them in nearby clinics were major barriers [32,34].

The mixed-methods design also provided important context for interpreting the survey findings. Although nearly all survey respondents reported regular internet use and online health information seeking, interview participants described substantial variation in broadband availability and digital literacy across their communities. This apparent discrepancy likely reflects characteristics of the study sample, since participation in the online survey required internet access, while interviews captured broader perceptions of community-level barriers. Similarly, interviews revealed informational gaps that were not fully apparent in the survey, including uncertainty regarding where genetic services are available, how testing is performed, and how results would influence medical care. These findings illustrate the complementary value of qualitative methods for identifying barriers that may not be readily captured through structured questionnaires.

Several limitations should be considered when interpreting these findings. An important limitation is the potential for selection bias, as all participants were already enrolled in ODG and therefore may have had greater interest in, familiarity with, or more favorable attitudes toward genetics research than rural individuals not enrolled in genetic research. The study population was predominantly White, older, and relatively well-educated, limiting generalizability to more diverse rural communities. In addition, recruitment required email and internet access, potentially excluding individuals with the greatest digital barriers. Although the qualitative sample was relatively small, thematic saturation was achieved for the principal domains explored.

Despite these limitations, this study provides important insights into factors that may influence successful implementation of genetics and precision medicine in rural populations. Among participants in this study, our findings suggest that implementation efforts should focus not only on willingness to engage with precision medicine, but also on reducing structural barriers, strengthening trust, and improving awareness of available services. Many of the barriers identified in this study, including cost, insurance coverage, and access to specialty care, are not unique to genetics and reflect broader challenges in accessing healthcare in rural communities; however, concerns regarding the privacy, potential misuse, and discriminatory use of genetic information may present additional considerations specific to the implementation of precision medicine. Educational initiatives should emphasize practical applications of genetics, utilize trusted local healthcare providers and community organizations, and provide culturally appropriate, plain-language communication. Importantly, because participants were already enrolled in a genetics research study, their generally favorable attitudes toward genetics and precision medicine may not reflect those of the broader rural population. Future studies should seek to confirm these findings in larger, more diverse rural populations that have not been preselected through participation in genetics research. Furthermore, research is needed to evaluate implementation strategies designed to improve access to genetic services, expand provider education in rural settings, and reduce financial and logistical barriers that may limit equitable participation in precision medicine. Collectively, these efforts will be essential to ensuring that the benefits of precision medicine are accessible to rural populations and do not inadvertently widen existing healthcare disparities.

## Figures and Tables

**Table 1:** Comparison of enrollment demographics between three cohorts and the US Census data. Table 1A shows cohort-level data (all participants); Table 1B shows only rural participants. Chi-square tests of independence were performed to compare demographic characteristics between ODG and the two other cohorts.

	OneDukeGen ^1^ (n = 10,305)	Duke Clinical Research Management System ^2^	All of Us Study ^3^	US Census Data (NC 2020)
A. This table includes all participants				
% Rural Participants	31.7%	19.1% ***	3.1% ***	21.4% ***
B. This table includes rural participants only				
Sex (Female)	51.2%	55.4% ***	70.2% ***	51.1%
Age (years) ^4^	18–24: 0.9% 25–34: 3.2% 35–59: 33.6% 60–74: 45.8% 75+: 16.6%	0–5: 5.0% 5–14: 3.4% 15–24: 5.0% 25–34: 7.4% 35–59: 33.4% 60–74: 35.6% 75+: 10.3% ***	Not available	0–5: 5.1% 5–14: 11.7% 15–24: 12.3% 25–34: 11.0% 35–59: 30.9% 60–74: 20.4% 75+: 8.7% ***
Race	White: 85.8% Black/African American: 9.6% Other/More than 1 race: 4.5%	White: 70.4% Black/African American: 23.1% Other/More than 1 race: 2.8% ***	White: 62.6% Black/African American: 13.9% Other/More than 1 race: 3.4% ***	White: 66.1% Black/African American: 19.7% Other/More than 1 race: 14.2% ***
Ethnicity (Hispanic)	2.1%	1.7%	14.8 ***	9.2% ***

^1^ Includes all ODG participants. ^2^ Data from https://doi.org/10.1111/cts.13885. ^3^ Data from https://doi.org/10.1111/jrh.12840. ^4^ For the age chi-square test, only participants age 25+ were included; participants age 0–24 years of age were removed due to categorical mismatch. ***: *p* < 0.001.

**Table 2:** Demographic characteristics of survey and interview participants.

	Invited to Participate (n = 517)	Completed Survey (n = 111)	Completed Interviews (n = 14)
Sex	49.3% Female	58.6% Female *	50.0% Female
Age (years)	62.0 (mean); 64.0 (median); 18–88 (range)	60.8 (mean); 63 (median); 18–88 (range)	61.3 (mean); 68 (median); 38.0–81.0 (range)
Race	86.8% White 2.5% Multiple Races 10.1% Black 0.6% Other, Asian, Filipino	87.4% White 6.3% Multiple Races 5.4% Black 0.9% Other	85.7% White 14.3% Multiple Races 0% Black 0% Other
Ethnicity	97.1% Not Hispanic	98.2% Not Hispanic	100% Not Hispanic
Education	77.6% Some college or higher degree 22.4% High-school graduate or less	85.6% Some college or higher degree 14.4% High-school graduate or less	71.4% Some college or higher degree 28.6% High-school graduate or less
Employment Status	46.6% Retired 29.0% Working full-time 13.9% Permanently Disabled 10.4% Other	43.2% Retired 29.7% Working full-time 13.5% Permanently Disabled 13.5% Other	50.0% Retired 21.4% Working full-time 14.3% Permanently Disabled 13.3% Other
Heath Status	4.8% Excellent 18.0% Very Good 41.8% Good 26.5% Fair 8.7% Poor	9.9% Excellent 17.1% Very Good 42.3% Good 23.4% Fair 7.2% Poor	14.3% Excellent 28.6% Very Good 42.9% Good 7.1% Fair 7.1% Poor
Health Insurance	Not Available	57.7% Medicare 10.8% Medicaid 7.2% Military (TriCare/VA) 8.1% Other state-sponsored health plan 37.8% Private insurance 1.8% Other govt program 3.6% Self-Insured	50.0% Medicare 14.3% Medicaid 7.1% Military (TriCare/VA) 14.3% Other state-sponsored health plan 42.9% Private insurance 0% Other govt program 0% Self-Insured
OneDukeGen Recruitment Method	60.3% Homekit 39.7% Onsite	82.0% Homekit 18.0% Onsite	92.9% Homekit 7.1% Onsite

* Significant (*p* < 0.05).

**Table 3:** Respondents’ reasons for delaying medical care (respondents could select more than one reason).

Reasons for Delay in Medical Care	Overall (n = 111) % (n)
Didn’t have transportation.	6.3% (7)
You live in a rural area where the distance to the health care provider is too far.	11.7% (13)
You were nervous about seeing a health care provider.	11.7% (13)
Couldn’t get time off work	7.2% (8)
Couldn’t get child care.	1.8% (2)
You provide care to an adult and could not leave him/her.	4.5% (5)
Couldn’t afford the copay.	8.1% (9)
Your deductible was too high/or could not afford the deductible.	7.2% (8)
You had to pay out of pocket for some or all of the procedure.	15.3% (17)

**Table 4:** Major themes by interview guide topics.

Interview Guide Topic	Dominant Themes
General awareness/attitudes	Optimism, fascination, future medical advances
Concerns/reservations	Privacy, misuse, discrimination, government overreach
Clinical applications	Personalized medicine, prevention, family risk awareness
Accessibility/barriers	Cost, insurance, lack of awareness, rural access
Information/support needs	Trusted providers, outreach, plain-language education
Rural-specific issues	Cultural distrust, church/community influence, digital divide

## Data Availability

De-identified raw data supporting the conclusions of this article will be made available by the authors on request.
